# ‘Cloud computing’ and clinical trials: report from an ECRIN workshop

**DOI:** 10.1186/s13063-015-0835-6

**Published:** 2015-07-29

**Authors:** Christian Ohmann, Steve Canham, Edgar Danielyan, Steve Robertshaw, Yannick Legré, Luca Clivio, Jacques Demotes

**Affiliations:** European Clinical Research Infrastructures Network (ECRIN), BioPark, 5-7 rue Watt, 75013 Paris, France; European Clinical Research Infrastructure Network (ECRIN), Prinz-Georg-Str. 51, 40477 Düsseldorf, Germany; Danielyan Consulting Ltd. Turnbull House, 226 Mulgrave Road, Sutton Surrey, SM2 6JT UK; xKavate Knowledge Mining Ltd., Fryern House, 125 Winchester Road, Chandlers Ford, Hampshire, SO53 2DR UK; European Grid Initiative (EGI), Science Park 140, 1098 XG Amsterdam, The Netherlands; Istituto di Recherche Farmacologische Mario Negri, Via La Mass 19, 20156 Milano, Italy

**Keywords:** Clinical trials, Cloud computing, Distributed computing, IT infrastructure, Security, Regulatory compliance

## Abstract

Growing use of cloud computing in clinical trials prompted the European Clinical Research Infrastructures Network, a European non-profit organisation established to support multinational clinical research, to organise a one-day workshop on the topic to clarify potential benefits and risks. The issues that arose in that workshop are summarised and include the following: the nature of cloud computing and the cloud computing industry; the risks in using cloud computing services now; the lack of explicit guidance on this subject, both generally and with reference to clinical trials; and some possible ways of reducing risks. There was particular interest in developing and using a European ‘community cloud’ specifically for academic clinical trial data. It was recognised that the day-long workshop was only the start of an ongoing process. Future discussion needs to include clarification of trial-specific regulatory requirements for cloud computing and involve representatives from the relevant regulatory bodies.

## Background

Recent years have seen rapidly growing interest in ‘cloud computing’, driven by the promise of cheap and flexible information technology (IT) infrastructure available as a service, reducing the requirements for in-house systems and staff. Trials units and vendors of clinical data management systems (CDMSs) are not immune to these temptations. Moreover, there are use cases in which cloud computing may be a good fit (for instance, the aggregation of data for meta-analysis or the long-term curation of trial data). Within ECRIN-IA, an EU Seventh Framework Programme (FP7)-funded project, the European Organisation for Research and Treatment of Cancer is developing VISTA Trials, based on an existing widely used CDMS. The current need to explore options for this system’s deployment serves as ECRIN’s own use case.

The difficulty is that cloud computing technology brings risks as well as benefits. We need a better understanding of both, and we need to engage regulators to clarify the criteria by which cloud computing services can be judged. ECRIN organised a one-day workshop (30 Oct. 2014, Brussels, Belgium) to instigate this process, and this commentary summarises the points that emerged that day.

## Main text

The commentary is based on a workshop. For that reason, no ethical approval was required and no informed consent obtained.

### What is ‘cloud computing’?

We see cloud computing as an extension of traditional outsourced IT provision, but confusion still surrounds what the term ‘cloud computing’ means. We use the National Institute of Standards and Technology definition [1]: user-managed service configuration, rapid scalability and elasticity, and a metered cost model.

The industry is growing very rapidly [2], exhibits fierce competition with (until very recently) rapidly decreasing costs [3], and is dominated by US-based companies, in particular Amazon (Seattle, WA, USA) and Microsoft Corporation (Redmond, WA, USA) but also Google (Mountain View, CA, USA) and IBM Corporation (Armonk, NY, USA) [4].

Though open or public clouds are the most common, it is possible to have cloud services limited to particular types of organisations (‘community clouds’) or a single organisation (a ‘private cloud’). There is growing interest in so-called ‘hybrid clouds’, denoting a mix of private and public or community cloud services.

### Possible risks

The meeting identified the following major risks in using cloud computing:

- *Lack of control*: Cloud computing involves plugging into pre-existing services rather than negotiating bespoke provision. Many services (e.g., the provision of redundant copies of data to maintain availability) are managed invisibly by the cloud provider. A trials unit may not control the countries in which its data is stored, even though data crossing international borders can generate additional legal and regulatory obligations. Data that a unit believes to have been deleted may still be retained somewhere by the cloud service [5]. Cloud users should be aware that cloud service providers may not be held contractually responsible for hosting poorly designed systems [6].
- *Industry volatility*: Fierce competition means that several cloud providers are likely to disappear in the next few years. It is not clear how this could affect subsequent access to data. Data lock-in makes it difficult for a customer to migrate from one provider to another or migrate data and services back in-house [7].
- *Security*: Cloud providers put considerable resources into security but are also obvious targets for those wishing to steal data. This no longer involves ‘amateur’ hacking; it is a sophisticated criminal activity, with trial data being viewed as having a re-sale value. In addition, in the cloud, it is not always clear who is responsible for different aspects of security. There is a risk of security controls ‘falling through the cracks’ if the customer is not fully aware of their role. The situation may get even more critical if clinical trials are linked to biobanks, and the question needs to be addressed how to handle the even more sensitive molecular data if anonymisation cannot be performed.
- *Suitability*: Individual clinical trial datasets rarely exceed a few gigabytes and are fixed once the trial is over. They are stored for archiving and audit rather than reuse and updating. There is a risk that the costs for cloud provisioning of such basic services could overtake the costs of delivering them via conventional means.
- *Uncertainties over legal jurisdictions*: As recent court cases have demonstrated [8], there is continued uncertainty about the legal frameworks that apply to cloud data. Data may be sited within and also move through—via routers, switches, and satellites—a variety of countries. In this context, a range of issues, most conspicuously the application of EU versus non-EU data access regulations, still await clarification.
- *Lack of knowledge*: Hosting offered by CDMS vendors and others for trial data increasingly involves cloud computing facilities. Hosts are often unclear, however, about the exact cloud computing arrangements, making it much more difficult for the unit to investigate and demonstrate regulatory compliance.

### Lack of compliance guidelines

Compounding the problems listed above is the lack of regulatory guidance. The cloud-specific International Organization for Standardization (ISO) standard (27017) currently at the FDIS (final draft international standard) approval stage (ISO 27018 has recently been approved). There are general ‘Service Organization Control’ standards in the US but these have limited applicability in Europe. Other systems being developed include the Cloud Security Alliance’s Security, Trust & Assurance Registry (STAR) system [9], but take-up of this appears limited.

At present, there appears to be little Good Clinical Practice-specific guidance on using cloud services. ECRIN is among the organizations that need to address this issue, reviewing their own standards for data centre certification [10] in the light of possible cloud computing usage.

### Reducing risks

The following were identified as possible ways of reducing risks:

- *Identify requirements*: It is important to be clear why a cloud computing solution is preferable to a traditional arrangement and to identify the specific requirements within that solution.
- *Assess options by using a risk-based approach*: All types of risks (technical, legal, and organisational) should be assessed to identify vulnerabilities, threats, and mitigations [7, 11].
- *Have explicit contracts*: Ideally, once requirements are identified, they should be incorporated into the purchased services through explicit contracts: providers who cannot provide and prove these specific requirements should be avoided. There should be an option that a customer or a third party may carry out tests and audits [11].
- *Use encryption at source*: Particularly for public clouds, one way of guaranteeing privacy is to encrypt all data ‘at source’ (i.e., under the control of the trials unit rather than the cloud provider). However, the systems, time, and money required for effective encryption key management should not be underestimated. There are also widely differing governmental attitudes toward the public use of encryption systems in the EU nations.
- *Develop European Community clouds*: Using community clouds (e.g., restricted to universities in Europe) would be one way of alleviating some of the security/control risks of public clouds. If they were based in and managed by European institutions, there would also be no question that they fell into a European jurisdiction for data protection purposes.
- In this context, there was considerable interest in using the ‘federated cloud’ approach of the European Grid Infrastructure (EGI) project [12], which makes use of a distinct physical infrastructure (Géant) [13] for communicating between nodes and is actively policed for security breaches. It was felt that a ‘mini-EGI’ established specifically for trial data could realise many of the benefits of cloud computing with reduced risk, although it was recognised that there would be many practical hurdles to overcome. Another innovative approach, described in the workshop, used a peer-to-peer network to share resources between trials units and clinical sites.

## Conclusions

The ECRIN workshop was a beginning; there were more questions raised than questions answered. Trials units need time to understand cloud computing and consider the risks and benefits. Technology is developing rapidly and becoming cheaper, more tempting, more widely used, and therefore possibly more dangerous. We urgently need to characterise the acceptable use of cloud computing for clinical trials. We also need to explore the options for developing a community cloud facility specifically for clinical trial data. ECRIN is keen to engage with other organisations and agencies in the pursuit of those goals. The next concrete step will be to perform a follow-up workshop with experienced clinical trial and IT experts and representatives from regulatory authorities, ethics committees, and data protection organisations with the target to clarify trial-specific regulatory requirements for cloud computing and to explore consequences for the ECRIN data centre certification programme.

## Abbreviations

CDMS: Clinical Data Management System
ECRIN: European Clinical Research Infrastructures Network
EGI: European Grid Initiative
IT: Information technology
